# *Molecules* after 20 Years—Looking Back and Looking Forward

**DOI:** 10.3390/molecules20010645

**Published:** 2015-01-06

**Authors:** Derek J. McPhee

**Affiliations:** Editor-in-Chief, MDPI AG, Klybeckstrasse 64, Basel CH-4057, Switzerland; E-Mail: mcphee@mdpi.com

Twenty years—a long time—so what happens after 20 years? Well if you live in New Zealand, according to [1], if you are adopted you can apply to Births, Deaths and Marriages for a copy of your birth certificate to find the names of your birth parents, you can apply to adopt a child who is related to you and you can gamble or work in a casino [1], but more seriously, our 20th anniversary is an appropriate time to look back at the history of *Molecules* and perhaps offer some speculations about its future.

To tell the history of *Molecules*, one must go back to the creation of MDPI in 1995, a little before its first issue appeared. That year Dr. Shu-Kun Lin, the company’s current President, who was working at the time at Ciba-Geigy in Basel, Switzerland, conceived the idea of a worldwide center for the collection and sharing of rare chemical samples as a way of preserving chemical diversity for the scientific record. To promote the project, an online journal of synthetic chemistry called *Molecules* was created and the corresponding ISSN 1420-3049 was obtained. An editorial board was set up with Dr. Lin as Editor-in-Chief and advertisements were published in some of the leading journals of the day to promote the samples collection project [2]. In 1996 Molecular Diversity Preservation International (MDPI) was officially founded and registered with the commercial register in Basel in June by Dr. Shu-Kun Lin and Dr. Benoit R. Turin as a non-profit institute. The institute would later become the current publishing house Multidisciplinary Digital Publishing Institute AG (MDPI AG). The samples project still exists—MDPI was dissolved in 2013 and the collection of samples was permanently transferred to the MDPI Sustainability Foundation, on whose behalf the collection is now operated by molMall Sarl [3].

Also in 1996 the scientific peer-reviewed journal *Molecules* became one of the first electronic chemistry journals when it was launched with a monthly frequency, in collaboration with Springer Verlag. The University of Basel hosted the first homepage for MDPI at www.unibas.ch/mdpi. Later, MDPI would launch its own server and the website was made available at www.mdpi.org. At the end of 1996, Springer Verlag terminated the agreement with MDPI and sought to continue publication of *Molecules* under its imprint, which led to a lawsuit between MDPI and Springer over ownership of the journal.

In 1997, after consultation among the members of the International Editorial Board, MDPI took back the publishing of *Molecules*, and Volume 2 was published under the direction of Dr. Esteban Pombo-Villar as Editor-in-Chief. The *Molbank* section of *Molecules* was introduced in the same year. *Molbank* publishes papers on synthetic compounds and natural products as one-compound-per-paper short notes; in 2003 the section became an independent journal with its own ISSN (1422-8599). I, the current Editor-in-Chief of *Molecules*, started working on a part time basis as Editorial Assistant to Dr. Pombo-Villar. Until 2002 when the first staffed Editorial Offices started operations, he would handle from Canada all the manuscript processing duties (manuscript receipt, peer review, issue layout and editing) for *Molecules* and some of MDPI’s new journals as they were launched, while Dr. Shu-Kun Lin handled all the actual publishing and administrative tasks.

In late 1998 the dispute between MDPI and Springer was resolved out of court. While MDPI has continued to publish *Molecules* under its original ISSN until this day, Springer Verlag started publication of a competing journal titled *Molecules Online* (ISSN 1433-1373), with Prof. Steven Hanessian as Editor-in-Chief. It was discontinued after two volumes in 1999.

In 1999 Dr. Shu-Kun Lin took over again as Editor-in-Chief of *Molecules*, replacing Dr. Pombo-Villar whose senior management duties with Novartis no longer allowed him to dedicate appropriate attention to the journal, and I was formally appointed Managing Editor. The start of the new millennium saw in 2000 the brief appearance if a print edition of *Molecules*. Under the direction of Dr. Francis Muguet and with the kind cooperation of the administration of the École Nationale Supérieure de Techniques Avancées in Paris (France), limited print editions of Volumes 5 and 6 of *Molecules*, and a few other MDPI journals were produced and distributed to several academic and industrial laboratories [4]. Also in 2002, *Molecules* prepared and hosted from 15–18 July a very successful international conference in Qingdao (China), the International Symposium on Frontiers in Molecular Science 2002 (ISFMS 2002); the journal’s first e-publishing system was set up and the first publishing office outside Switzerland was set up in Qingdao, China with the sponsorship of Ocean University of China [5].

For the next five years the journal’s steady growth continued. In 2005, the same year the journal celebrated its 10th anniversary [6], I accepted the appointment as Editor-in-Chief of *Molecules*, while Dr. Shu-Kun Lin started holding the title of Publisher and Director of the Molecules Editorial Office. During 2005–2006 papers were published in both non-Open Access (non-OA) and Open Access (OA) form, and since 2007 *Molecules* has published papers exclusively in Open Access form. In 2008 *Molecules* started adding the Digital Object Identifier (DOI) numbers to each paper using the CrossRef system, the Open Access logo was also added and the copyright line was moved to Creative Commons (CC) “By Attribution” license v.3.0 model. In March 2014 *Molecules* published its 6000th paper. We would close out our 19th year having published 1301 papers.

So what does the future hold for us beyond our 20th anniversary in 2015? To mark the occasion we will be formally announcing in the coming months the addition of a series of Associate Editors selected from among the many world famous scientists who have been active in *Molecules* as Guest editors of Special Issues during the past 20 years, and have shown an interest in the success of the journal. They will be helping with the ever expanding workload of running a major global journal, and we anticipate expanding our sponsorship of some major international conferences and our collaboration with select academic institutions.

As we start this important milestone year, I must offer our sincere thanks to our readers and the innumerable authors, anonymous peer reviewers, Assistant Editors at our Editorial Offices in Switzerland and China, and English language editors who have contributed to each and every one of our papers. Without all of you this fantastic journey would never have been possible.
